# Birth Preparedness and Complication Readiness Among Pregnant Women: A Facility-Based Cross-Sectional Study

**DOI:** 10.1016/j.focus.2025.100374

**Published:** 2025-06-06

**Authors:** Matilda Adu-Gyamfi, Prince Owusu Adoma, Francis Acquah, Elvis Enowbeyang Tarkang

**Affiliations:** 1 Department of Population and Behavioural Sciences, School of Public Health, University of Health and Allied Sciences, Ho, Ghana; 2 Department of Health Administration and Education, Faculty of Health, Allied Science and Home Economic Education, University of Education, Winneba, Ghana; 3 HIV/AIDS Prevention Research Network Cameroon, Kumba, Cameroon; 4 Department of Public Health Medicine, School of Nursing and Public Health, University of KwaZulu-Natal, Durban, South Africa

**Keywords:** Birth preparedness and complication readiness, pregnant women, Cape Coast, Ghana

## Abstract

**Introduction:**

Birth preparedness and complication readiness have the goal of reducing maternal and neonatal mortality. This concept requires pregnant women to seek health care without delay in case of obstetric complications and delivery. This study determined the practices and factors associated with birth preparedness and complication readiness among pregnant women in the Cape Coast Metropolis, Ghana.

**Methods:**

A facility-based cross-sectional design was employed among 384 participants, collecting data using a self-administered questionnaire and analyzing them using Stata (Version 16) at the 0.05 significance level and 95% CI.

**Results:**

The practice of birth preparedness and complication readiness was 53.4%, and single pregnant women were more likely to practice birth preparedness and complication readiness (AOR=2.19; 95% CI=1.13, 3.45; *p*=0.020). Respondents whose husbands had completed the tertiary education level were more likely to practice birth preparedness and complication readiness (AOR=1.15; 95% CI=0.05, 1.46; *p*<0.001). Pregnant women who did not have enough money to cater for expenses, those who had no means of transportation to the health facility, those who did not have someone to accompany them to the health facility, those who did not receive advice from a birth attendant regarding their pregnancy, and those who did not have enough food at home to cater for the household were less likely to practice birth preparedness and complication readiness (all: *p*<0.05).

**Conclusions:**

Health promotion programs to increase the practice of birth preparedness and complication readiness should target all women, particularly those with limited social and financial support and families with no or basic education.

## Highlights

• Practices for birth preparedness and complication readiness were good.
• Birth preparedness and complication readiness practices differ by aspects.
• Association existed between husband’s education and birth preparedness and complication readiness.
• Pregnant women had funds available for transportation to the hospital.

## INTRODUCTION

Maternal and neonatal mortality remains a significant problem in developing countries, including Ghana.^1^ This is because 1 in 180 pregnant women die during childbirth compared with 1 in 4,900 in developed countries. About 75% of maternal deaths occur as a result of complications during pregnancy and childbirth.^2^ Birth preparedness and complication readiness (BPCR) are an effective strategy to prevent maternal mortality. This strategy encourages pregnant women to promptly seek care from skilled birth attendants (SBAs).^1^^,^^3^ With the presence of an SBA, the possibility of death due to intrapartum-related complications or stillbirth can be reduced by 20%.^4^

Care seeking is delayed because of the delay in (1) identifying the complications, (2) deciding to seek care, (3) identifying and reaching a health facility, and (4) receiving adequate and appropriate treatment.^5^ In many societies, cultural beliefs and a lack of awareness inhibit the preparation for delivery and seeking care.^3^ Owing to these reasons, complications in an unprepared family could take lots of time to understand, and family could take lots of time to get organized, get money, find transport, and reach the appropriate referral facility. The delay in reaching the health facility and receiving care can be solved by properly using the BPCR plan.^5^

If most of the complications that lead to maternal deaths is treated on time, maternal mortality could be significantly reduced.^3^ The key elements needed for the preparation of birth include knowledge of danger signs, a plan for where to give birth, a plan for a birth attendant, a plan for transportation, and a plan for saving money. In addition, a potential blood donor must also be identified.^5^ Inadequate knowledge of BPCR among expecting couples delays timely access to maternal emergency services.^5^ Poor knowledge about obstetric danger signs and what to prepare for childbirth influences the total level of a family’s BPCR.^6^ Increasing knowledge about safe motherhood practices should translate into safer pregnancy outcomes and lead to lower maternal mortality across the developing world.^7^

Globally, 810 women die every day owing to pregnancy or childbirth-related complications.^8^ Almost all maternal deaths (99%) occur in developing countries, and more than half of these deaths occur in sub-Saharan Africa.^8^ This is because of several reasons; one of which is the inadequacy or lack of birth and emergency preparedness, a key component of globally accepted safe motherhood programs.^9^

The government of Ghana has put interventions in place to curb maternal mortality. These interventions include the free maternal health and safe motherhood initiative. Despite these efforts, Ghana still records a maternal mortality of 308 per 100,000 live births.^10^ Although the benefits of BPCR have been documented in several countries^1^^,^^5^^,^^6^ and Ghana,^11^^,^^12^ little or no information is currently available on the practice of BPCR in the Central Region, where the prevalence of maternal death is relatively higher than the national average.^10^ Owing to this high prevalence of maternal deaths in the Central Region of Ghana, it is therefore important for this study to be conducted there. Again, attention has not been drawn to the effective implementation of BPCR because it is not routinely assessed in Ghana. Therefore, this study seeks to determine the practices of BPCR and associated factors among pregnant women in the Cape Coast Metropolis, Central Region of Ghana.

## METHODS

### Study Sample

The population of the Cape Coast Metropolis, according to the 2010 Population and Housing Census,^13^ is 169,894, representing 7.7% of the region’s total population. Males constitute 48.7%, and females represent 51.3%. A total of 23% of the population live in rural localities. The metropolis has a sex ratio (number of males per 100 females) of 95% males to 100% females. The proportion of the metropolis youth (aged <15 years) is 28.4%, depicting a not-too-broad-based population pyramid that tapers off with a small number of elderly (aged ≥60 years) persons (4.5%). The total age dependency ratio for the metropolis is 49.1, and the age dependency ratio for males is lower (48.2) than that of females (49.9). The metropolis has a regional hospital, a district hospital, and various clinics that provide health care to the population. The regional hospital, 1 of 3 such facilities in the country, serves as a regional referral center.

The study employed a facility-based cross-sectional design conducted in April 2021. Cross-sectional studies provide a snapshot of the outcome and its associated characteristics at a particular time.

The study population involved pregnant women between the ages of 14 and 40 years who had lived in Cape Coast for at least 6 months. Pregnant women between the ages of 14 and 40 years and gave their consent were included in the study. Parental consent was obtained for participants who were aged <18 years before they were made to participate in the study. However, participants who were severely ill during the study were excluded.

Multistage sampling was used in this study. Three health facilities providing antenatal care (ANC) services were randomly selected in the first stage. This was done by writing the names of all the facilities on pieces of paper, wrapping them, and placing them in a container. Three facilities were picked without replacement from the container. At the selected facilities, participants who met the eligibility criteria and consented to participate in the study were conveniently selected. Even though the convenience sampling method lacks generalization, it is the most readily available and convenient method to collect data among pregnant women. The convenience sampling method made it relatively easier to recruit all pregnant women who met the inclusion criteria for the study. Mothers and pregnant women who visited the ANC unit at their expected date were conveniently sampled. In this study, 384 pregnant women were conveniently sampled. This sample size was derived using the Yamane (1967) formula^14^:

$$n=N/[1+N{\left(e\right)}^{2}]$$

Where n is the sample size, N is the population under study, e is the proportion of sampling error put at 0.05 level of significance, 1 is the constant, N is 10,000; and e is 0.05. It should be noted that the choice of a 0.05 level of significance (i.e., 95% confidence limit) is purely an executive decision of the researcher. Substituting the above values into the formula, the following sample size was calculated:

$$n=10,000/(1+10,000{\left[0.05\right]}^{2})$$

n=10,000/(1+10,000[0.0025])

n=10,000/(1+25)

n=10,000/(26)

n˃384.615 (rounded down to the whole number)=384. Therefore, a sample size of 384 pregnant women was selected for this study.

Data were collected using a structured questionnaire designed in the English language. However, questions were explained in English and the local dialect (Fante). This ensured a better understanding of the questions. The questionnaire was adapted from previous studies.^3^^,^^9^^,^^11^^,^^15^^,^^16^ It consisted of 3 sections: A, B, and C, where A captured data on the participant’s demographic characteristics, B captured data on the practices of BPCR, and C captured information on the factors associated with BPCR. Two research assistants were trained to help in the data collection. A pretest was conducted on 10 pregnant women excluded from the actual study to verify the validity and reliability of the questionnaire.

### Measures

The questions used to measure the practice of BPCR were recorded as *No* for poor practice and *Yes* for good practice. After summing up all questions under practice, the average score was used to determine the composite score of poor and good practice. To determine the percentages of *Yes* and *No* for each question, the authors added the *Yes* and *No* responses from each participant and divided that amount by the number of participants. Regarding the practice of BPCR by pregnant women, 11 items were used. Pregnant women who answered *yes* were said to have a good practice of BPCR, and those who answered *no* were considered to have a poor practice of BPCR.

Furthermore, 6 questions were used to assess the factors of BPCR. However, in this case, respondents who answered *yes* were said to have some of the factors associated with their BPCR, and those who answered *no* were considered not to have some factors related to BPCR.

### Statistical Analysis

The collected data were checked for completeness and numbered serially; they were then entered into SPSS (Version 24.0) for statistical analysis. Data were screened for missing values for continuous and string variables, and univariate and multivariate outliers were checked using frequency distributions and box plots. The data were normally distributed, and box plots revealed that the data were homogenous with few outliers. All numerical data were analyzed using descriptive statistics. A binary logistic regression using 3 models was employed to determine the factors influencing birth BPCR. The first model comprised demographic variables, whereas the second model comprised other factors. The final model integrated significant factors to determine specific factors influencing BPCR. Only *p*-values <0.05 were considered statistically significant at a 95% CI. Tables are used to present the results.

## RESULTS

In terms of sociodemographic characteristics of respondents, the majority were aged between 20 and 25 years (116, 30.2%), were married (187, 48.7%), had gravidity of 2 (131, 34.1%), were Christians (300, 78.1%), were self-employed (195, 50.8%), had a senior high-school certificate as their highest educational level (149, 38.8%), were currently living with their partners (284, 74.0%), had husbands with a tertiary level of education (168, 43.8%), and were living in the urban area (281, 73.2%) (Table 1).

**Table 1:** Sociodemographic Characteristics of Respondents

Variables	Frequency (*n*=384)	Percentage (100%)
Age group, years		
14–19	56	14.6
20–25	116	30.2
26–30	104	27.1
31–35	92	23.9
36–39	16	4.2
Marital status		
Married	187	48.7
Single	183	47.7
Others	14	3.6
Gravidity		
1	171	44.5
2	131	34.1
3	60	15.6
≥4	22	5.7
Parity		
1	147	58.1
2	61	24.1
3	17	6.7
4	28	11.2
Religion		
Christian	300	78.1
Others	84	21.9
Respondent’s occupation		
Government employed	68	17.7
Self-employed	195	50.8
Unemployed	121	31.5
Highest educational level		
No education	9	2.3
Basic education	49	12.8
JHS	108	28.1
SHS/SSS	149	38.8
Tertiary	69	18.0
Currently living with your partner		
Yes	284	74.0
No	100	26.0
Husband's educational level		
Basic education	37	9.6
Secondary	168	43.8
Tertiary	136	35.4
No education	28	7.3
JHS	15	3.9
Area of residence		
Urban	281	73.2
Peri-urban	19	5.0
Rural	84	21.8

JHS, junior high school; SHS, senior high school; SSS, senior secondary school

### Aspects of Birth Preparedness and Complication Readiness

In terms of aspects of birth preparedness and complication readiness, from Table 2, the majority (227, 59.1%) of the pregnant women had enough money to cater for expenses in buying items for delivery. The majority (322, 83.9%) had means of transportation to the health facility. Most (283, 73.7%) had their preferred health facility in mind. The majority (370, 96.4%) had someone to accompany them to the health facility during labor. The majority (324, 84.4%) received advice from a birth attendant regarding their pregnancy, and most (306, 79.7%) had enough food in the house to cater for the people in their household.

**Table 2:** Aspects of Birth Preparedness and Complication Readiness

Variables	Frequency (*n*=384)	Percentage (%)
Do you have enough money to cover the expenses of buying items for delivery?		
Yes	227	59.1
No	157	40.9
Do you have means of transportation to the health facility?		
Yes	322	83.9
No	62	16.1
Do you have any preferred health facilities in mind?		
Yes	283	73.7
No	101	26.3
Do you have anyone to accompany you to the health facility during labor?		
Yes	370	96.4
No	14	3.6
Have you received any advice from a birth attendant regarding your pregnancy?		
Yes	324	84.4
No	60	15.6
Do you have enough food in the house to cater for the people in your household?		
Yes	306	79.7
No	78	20.3

Concerning the practice of BPCR, the majority (266, 69.3%) had been engaging in some form of physical activity, most (206, 53.6%) were not taking any herbal concoctions apart from the medications given to them at the hospital, the majority (292, 76.0%) had funds available for transportation to the hospital, most (303, 78.9%) had identified the mode of transportation to the hospital during labor, the majority (319, 83.1%) had essential items needed for delivery, most (211, 54.9%) had not arranged for any blood donor, and the majority (306, 79.7%) had packed items required for the delivery and the baby. Concerning the identification of an SBA, the majority (273, 71.1%) said that they had identified one, the majority (338, 88.0%) complied with the medications given to them at the health facility, and most (324, 84.4%) ate food recommended by the health providers (Table 3).

**Table 3:** Practice of Birth Preparedness and Complication Readiness

Variables	Frequency (*n*=384)	Percentage (%)
Have you been engaging in any form of physical activity?		
Yes	266	69.3
No	118	30.7
Do you take any herbal concoctions apart from the medication given to you at the hospital?		
Yes	178	46.4
No	206	53.6
Have you made funds available for transportation to the hospital?		
Yes	292	76.0
No	92	24.0
Have you identified the mode of transportation to the hospital when labour begins?		
Yes	303	78.9
No	81	21.1
Do you have the essential items needed for delivery?		
Yes	319	83.0
No	65	17.0
Have you arranged for a blood donor?		
Yes	173	45.1
No	211	54.9
Have you identified a companion to take you to the hospital when in labour?		
Yes	355	92.5
No	29	7.5
Have you started packing your bag with the items needed for the delivery and the baby?		
Yes	306	79.7
No	78	20.3
Have you identified a skilled birth attendant?		
Yes	273	71.1
No	111	28.9
Do you comply with the medications given to you at the health facility?		
Yes	338	88.0
No	46	12.0
Do you eat the foods recommended by the health provider?		
Yes	324	84.4
No	60	15.6

*Note:* The majority of the respondents (53.4%) had a good practice of birth preparedness and complication readiness.

In terms of the association between factors and the practice of birth preparedness and complication readiness, single pregnant women were more likely to practice BPCR (AOR=2.19; 95% CI=1.13, 3.45; *p*=0.020). Respondents whose husbands had completed tertiary education level were more likely to practice BPCR (AOR=1.15; 95% CI=0.05, 1.46; *p*<0.001). Pregnant women who did not have enough money for expenses were less likely to practice BPCR (AOR=0.09; 95% CI=0.05, 0.18; *p*<0.001). Respondents who had no transportation to the health facility were less likely to practice BPCR (AOR=0.07; 95% CI=0.33, 0.50; *p*=0.004). In addition, pregnant women who did not have their preferred facility in mind were less likely to practice BPCR (AOR=0.29; 95% CI=0.14, 0.44; *p*<0.001), those who were not having someone to accompany them to the health facility during labor were less likely to practice BPCR (AOR=0.11; 95% CI=0.07, 0.53; *p*<0.001), those who had not received any advice from a birth attendant regarding pregnancy were less likely to practice BPCR (AOR=0.16; 95% CI=0.14, 0.49; *p*<0.001), and those who were not having enough food in the house to cater for the people in their household were less likely to practice BPCR (AOR=0.06; 95% CI=0.01, 0.44; *p*=0.006) (Table 4).

**Table 4:** Association Between Factors and Practice of Birth Preparedness and Complication Readiness

Factors		Practice		(X^2^) (*p*-value)	COR (95% CI) (*p*-value	AOR (95% CI) (*p-*value)
		Good *n* (%)	Poor *n* (%)			
Marital status						
Married		120 (60.9)	67 (35.0)		ref	ref
Single		69 (35.0)	114 (61.0)	28.85 (≤0.001)	3.00 (1.93, 4.51), <0.001	2.19 (1.13, 4.24), 0.020
Others		8 (4.9)	9 (4.0)		2.58 (0.25, 28.27), 0.379	0.935 (0.06, 22.51), 0.645
Husband's educational level						
Basic education		10 (5.1)	27 (14.4)		ref	ref
Secondary		77 (39.1)	91 (48.7)		0.44 (0.20, 0.96), 0.039	0.39 (0.14, 1.08), 0.070
Tertiary		91 (46.2)	45 (24.1)	225.04 (≤0.001)	0.18 (0.81, 0.41), <0.001	1.15 (0.05, 1.46), <0.001
No education		13 (6.6)	15 (8.0)		0.43 (0.15, 1.21), 0.109	0.28 (0.06, 1.25), 0.095
JHS		6 (3.0)	9 (4.8)		0.56 (0.16, 1.20), 0.361	0.27 (0.06, 1.33), 0.109
Have enough money to cater for expenses						
	Yes	167 (67.1)	27 (26.5)		ref	ref
	No	82 (32.9)	75 (73.5)	48.00 (0.000)	0.11 (0.06, 0.30) 0.000	0.09 (0.05, 0.18) <0.001
Have transportation to the health facility						
	Yes	258 (87.5)	64 (71.9)		ref	ref
	No	37 (12.5)	25 (28.1)	12.21 (0.000)	0.080 (0.56, 0.99) 0.001	0.07 (0.33, 0.50) 0.004
Have a preferred health facility in mind						
	Yes	166 (71.2)	117 (77.5)		ref	ref
	No	67 (28.8)	34 (44.5)	24.50 (0.000)	0.26 (0.15, 0.45) 0.000	0.29 (0.14, 0.44) <0.001
Have someone accompany you to the health facility during labour						
	Yes	281 (96.9)	89 (94.7)		ref	ref
	No	9 (3.1)	5 (5.3)	41.11 (0.000)	0.13 (0.06, 0.26) 0.000	0.11 (0.07, 0.53) <0.001
Have received some advice from a birth attendant regarding your pregnancy						
	Yes	210 (86.4)	114 (80.9)		ref	ref
	No	33 (13.6)	27 (19.1)	28.72 (0.000)	0.29 (0.18, 0.70) 0.000	0.16 (0.14, 0.49) <0.001
Have enough food in the house to cater for the people in your household						
	Yes	197 (75.8)	109 (87.9)		ref	ref
	No	63 (24.2)	15 (12.1)	18.69 (0.000)	0.55 (0.01, 0.41) 0.005	0.06 (0.01, 0.44) 0.006

COR, crude odds ratio; JHS, junior high school.

## DISCUSSION

In this study, the proportion of BPCR practice was 53.4%. This is higher than the levels reported in similar studies conducted in rural areas in Cameroon,^3^ Ghana,^11^ Ethiopia,^9^ and Tanzania.^17^ The differences between the findings of this study and those of the other studies could be due to economic factors. These studies were all conducted in rural areas, which are likely to experience wide socioeconomic developmental gaps compare with the study site of this study. However, the proportion reported in this study is lower than what was reported in a study conducted in urban areas in Greater Accra, Ghana (78%)^12^; in Osogbo metropolis of Southwest Nigeria (82.1%)^16^; and in an urban hospital in India (71.5%).^15^ The fact that some of the health facilities involved in this study were found in the rural areas of the Cape Coast Metropolis could account for the discrepancy between the current findings and those of these other studies.

In this study, some aspects of BPCR were more planned than others. For instance, this study reported that the majority of the pregnant women had funds available for transportation to the hospital and had identified the mode of transportation to the hospital during labor (76.0% and 78.9%, respectively). This contradicts the finding of a similar study conducted in Egypt^18^ but agrees with that of a study conducted in Ethiopia.^19^ Complications in pregnancy and delivery among mothers and pregnant women are primarily a result of delays in reaching health facilities for proper treatment. These delays are primarily due to the lack or inaccessibility of means of transport.^5^ This study's findings depict and confirm that urbanization reduces the rate of delays in reaching health facilities due to the availability of means of transport and monetary support for transportation. Good preparedness and readiness comprise the available means of transport and the ability to afford transportation costs to and from health facilities. This translates into a high level of preparedness and readiness among pregnant women. Several studies^20^, ^21^, ^22^ have associated pregnancy complications and maternal deaths with delays in reaching health facilities for maternal care and delivery. Making plans for transportation and being able to afford it will therefore reduce maternal mortality.

Furthermore, this study found that 83.1% of pregnant women had an essential item needed for delivery, and this contradicts the findings of a similar study conducted in Egypt,^18^ which found that 40% had essential items for delivery and newborn care. Essential items readiness depicts a psychological readiness and happiness for the baby, which might increase the strength during delivery and the hope of seeing the unborn child. Planning for essential items reduces stress and pressure during delivery, which comes from health workers.

Concerning this study, 71.1% identified an SBA, and this disagrees with the finding of a similar study conducted in Egypt,^18^ which revealed that 7.3% of respondents had identified a skilled provider for delivery. A high level of awareness of the dangers and consequences of home delivery without a birth attendant is likely to translate to looking for a birth attendant during pregnancy among pregnant women.

The least reported aspect of BPCR in this study was arranging for a blood donor. Most participants (54.9%) had not arranged for any blood donor, which is similar to the findings of a study conducted in Southwest Nigeria.^16^ This aspect might have contributed to the low practice of BPCR (53.4%) reported in this study. Generally, sociocultural perceptions concerning blood donation and reception in Ghana might deter some people from such practices^23^ despite high levels of iron-deficiency anemia, which is associated with postpartum hemorrhage, especially among pregnant women.^24^ Therefore, the government of Ghana should aim to improve national blood banking systems and promote blood donation. Blood transfusion should also be discussed with pregnant women early in the pregnancy to address misconceptions.^25^

Marital status and husband’s educational level were statistically significantly associated with the practices of BPCR. Single pregnant women were more likely to practice BPCR. This finding contradicts that of a study conducted in Nepal,^26^ which found that respondents who were married had better preparedness. Single mothers tend to be free from second-party decisions or influence and are also nondependent on a partner for financial support. The public health implication of the current finding is that because married women are not fully prepared for birth and ready for complications due to some reasons, which include being overburdened with house chores and taking care of the family, there would be an increase in complications and maternal mortality among them. It is recommended that educational programs on male involvement in BPCR should be emphasized. Emphasis should also be laid on the need for social support from friends and families to support married pregnant women.

Those whose husbands had a tertiary level of education were more likely to practice BPCR. This finding is similar to that of a study conducted in Ethiopia,^27^ which indicated that women whose husbands have a secondary education are more likely to be prepared than those whose husbands are illiterates. This implies that women whose partners have a higher educational background will better understand the concept of preparedness and thus influence their decisions. Husbands being the head of the family and sometimes having the final say is critical in making important health-related decisions, most especially when educated and informed.

The findings from this study revealed that pregnant women who had not received any advice from a birth attendant regarding pregnancy were less likely to practice BPCR than those who had received some advice from a birth attendant regarding pregnancy. This is consistent with the findings of a similar study conducted in Northwest Ethiopia^28^ and in North Ethiopia,^29^ which found that women who were advised on BPCR were more likely to practice it during labor and delivery. These findings reiterate the importance of health professionals in individuals’ decisions in accessing health care, which translates to quality healthcare delivery and, in this regard, reduced infant and maternal mortality. Therefore, it is incumbent on health professionals, especially nurses and midwives in this context, to give education that is from credible and authentic sources. Information and awareness creation should also be culturally friendly and easy to understand and practiced by pregnant women and mothers.

In addition, from this study, pregnant women who did not have enough money for expenses were likely to practice BPCR than those who had the money for costs, and this is consistent with findings of a similar study conducted in Ethiopia,^30^ which revealed that women having the highest wealth assets are more likely to practice BPCR than women in the lowest quartile. A study in India^31^ also showed that pregnant women in the highest quartile will have the means to save money for emergency funding and transportation. In addition, from this study, respondents who had no transportation to the health facility were less likely to practice BPCR than those who had transportation to the health facility. This is consistent with a study conducted in India,^31^ which also revealed that pregnant women in the highest quartile will have the means to save money for transportation.

In communities that have difficulties with transportation, the government should make available ambulances and vehicles that transport pregnant women to health facilities in emergencies. More health facilities and community-based health planning and services should be distributed across the country, and they should provide maternal and child health services, including ANC, delivery, and postnatal care services. This will help give equal chances of delivery success to financially challenged individuals and those facing transportation issues.

### Limitations

This study's findings should be interpreted in light of some limitations. First, being a cross-sectional study, causal relationships could not be ascertained. Second, the convenience sampling used in the last stage of the multistage sampling limits generalization of the findings.

## CONCLUSIONS

Pregnant women had an adequate practice of birth preparedness and complication readiness. The majority had funds available for transportation to the hospital, identified the mode of transportation to the hospital during labor, identified essential items needed for delivery, and identified an SBA. Marital status and husband’s educational level were statistically significantly associated with birth preparedness and complication readiness.

## References

[1] Kiataphiwasu N., Kaewkiattikun K. (2018). Birth preparedness and complication readiness among pregnant women attending antenatal care at the Faculty of Medicine Vajira Hospital, Thailand. Int J Womens Health.

[2] Apanga P.A., Awoonor-Williams JK. (2018). Maternal death in rural Ghana: a case study in the Upper East Region of Ghana. Front Public Health.

[3] Ijang Y.P., Cumber S.N.N., Nkfusai C.N., Venyuy M.A., Bede F., Tebeu PM. (2019). Awareness and practice of birth preparedness and complication readiness among pregnant women in the Bamenda Health District, Cameroon. BMC Pregnancy Childbirth.

[4] Ameyaw E.K., Dickson KS. (2020). Skilled birth attendance in Sierra Leone, Niger, and Mali: analysis of demographic and health surveys. BMC Public Health.

[5] Berhe A.K., Muche A.A., Fekadu G.A., Kassa GM. (2018). Birth preparedness and complication readiness among pregnant women in Ethiopia: a systematic review and meta-analysis. Reprod Health.

[6] Moshi F.V., Ernest A., Fabian F., Kibusi SM. (2018). Knowledge on birth preparedness and complication readiness among expecting couples in rural Tanzania: differences by sex cross-sectional study. PLoS One.

[7] Okereke E., Aradeon S., Akerele A., Tanko M., Yisa I., Obonyo B. (2013). Knowledge of safe motherhood among women in rural communities in northern Nigeria: implications for maternal mortality reduction. Reprod Health.

[8] WHO (2019). https://www.who.int/news-room/fact-sheets/detail/maternal-mortality.

[9] Hailu M., Gebremariam A., Alemseged F., Deribe K. (2011). Birth preparedness and complication readiness among pregnant women in Southern Ethiopia. PLoS One.

[10] Ghana Statistical Service, Ghana Health Service, Macro ICF. Ghana Maternal Health Survey 2017; Key Indicators Report. Accra: Ghana: Ghana Statistical Service, Ghana Health Service, and ICF; 2018.https://www2.statsghana.gov.gh/docfiles/PR95.pdf. Accessed March 27, 2024.

[11] Saaka M., Alhassan L. (2021). Prevalence and predictors of birth preparedness and complication readiness in the Kassena-Nankana district of Ghana: an analytical cross-sectional study. BMJ Open.

[12] Klobodu C., Milliron B.J., Agyabeng K., Akweongo P., Adomah-Afari A. (2020). Maternal birth preparedness and complication readiness in the Greater Accra region of Ghana: a cross-sectional study of two urban health facilities. BMC Pregnancy Childbirth.

[13] Ghana Statistical Service. Population and Housing Census: Summary Report of Final Results 2010. Cape Coast: Metropolis. Ghana Statistical Service, Accra, Ghana; 2010. https://www.statsghana.gov.gh/gssmain/storage/img/marqueeupdater/Census2010_Summary_report_of_final_results.pdf. Accessed April 9, 2024.

[14] Yamane T. (1967).

[15] Kamineni V., Murki A.D., Kota VL. (2017). Birth preparedness and complication readiness in pregnant women attending urban tertiary care hospital. J Family Med Prim Care.

[16] Sabageh A.O., Adeoye O.A., Adeomi A.A., Sabageh D., Adejimi AA. (2017). Birth preparedness and complication readiness among pregnant women in Osogbo Metropolis, Southwest Nigeria. Pan Afr Med J.

[17] Urassa D.P., Pembe A.B., Mganga F. (2012). Birth preparedness and complication readiness among women in Mpwapwa district, Tanzania. Tanzan J Health Res.

[18] Aziz M.M., Shams El-Deen R.M., Allithy MA. (2020). Birth preparedness and complication readiness among antenatal care clients in Upper Egypt. Sex Reprod Healthc.

[19] Markos D., Bogale D. (2014). Birth preparedness and complication readiness among women of child bearing age group in Goba woreda, Oromia region, Ethiopia. BMC Pregnancy Childbirth.

[20] Fiagbe P., Asamoah D., Oduro FT. (2012). Assessing the role of transport in the achievement of maternal mortality reduction in Ghana. Int J Bus Manag.

[21] Goodman D.M., Srofenyoh E.K., Olufolabi A.J., Kim S.M., Owen MD. (2017). The third delay: understanding waiting time for obstetric referrals at a large regional hospital in Ghana. BMC Pregnancy Childbirth.

[22] Adika D.M., Chutiyami M., Dathini H., Adamu H. (2017). Maternal mortality in Ghana: an exploration of partners’ perception about factors that contributed to their wife’s death. Int J Community Med Public Health.

[23] Asamoah-Akuoko L., Hassall O.W., Bates I., Ullum H. (2017). Blood donors’ perceptions, motivators and deterrents in sub-Saharan Africa – a scoping review of evidence. Br J Haematol.

[24] SPRING (2016).

[25] Schantz-Dunn J., M N. (2011). The use of blood in obstetrics and gynecology in the developing world. Rev Obstet Gynecol.

[26] Silwal K., Poudyal J.K., Shah R. (2020). Factors influencing birth preparedness in Rapti Municipality of Chitwan, Nepal. Int J Pediatr.

[27] Bitew Y., Awoke W., Chekol S. (2016). Birth preparedness and complication readiness practice and associated factors among pregnant women, Northwest Ethiopia. Int Sch Res Notices.

[28] Debelie T.Z., Abdo A.A., Anteneh K.T. (2021). Birth preparedness and complication readiness practice and associated factors among pregnant women in Northwest Ethiopia: 2018. PLoS One.

[29] Hiluf M., Fantahun M. (2008). Birth preparedness and complication readiness among women in Adigrat town, north Ethiopia. Ethiop J Health Dev.

[30] Azeze G.A., Mokonnon T.M., Kercho MW. (2019). Birth preparedness and complication readiness practice and influencing factors among women in Sodo town, Wolaita zone, southern Ethiopia, 2018; community based cross-sectional study. Reprod Health.

[31] Akshaya K.M., Shivalli S. (2017). Birth preparedness and complication readiness among the women beneficiaries of selected rural primary health centers of Dakshina Kannada district, Karnataka, India. PLoS One.

